# First synthesis of 2-(benzofuran-2-yl)-6,7-methylene dioxyquinoline-3-carboxylic acid derivatives

**DOI:** 10.3762/bjoc.7.28

**Published:** 2011-02-15

**Authors:** Wentao Gao, Jia Liu, Yun Jiang, Yang Li

**Affiliations:** 1 Institute of Superfine Chemicals, Bohai University, Jinzhou 121000, China

**Keywords:** benzofuran, Friedländer condensation, methylenedioxy-bearing, one-pot, quinoline-3-carboxylic acid, salicylaldehyde

## Abstract

A facile and inexpensive synthesis of a series of novel methylenedioxy-bearing 2-(benzofuran-2-yl)-quinoline-3-carboxylic acid derivatives **3a**–**h** via the one-pot reaction of ethyl 2-chloromethyl-6,7-methylenedioxyquinoline-3-carboxylate (**5**) with various substituted salicylaldehydes **6a**–**g** as well as 2-hydroxy-1-naphthaldehyde (**6h**) is described. Substrate **5** was synthesized by the Friedländer condensation reaction of 2-amino-4,5-methylenedioxybenzaldehyde (**4**) with ethyl 4-chloro-3-oxobutanoate using KHSO_4_ as catalyst under ultrasound irradiation conditions. The targeted compounds **3a**–**h** were obtained in good yields of 52–82% and their structures were established based on spectral data and elemental analyses.

## Introduction

**Figure 1:**
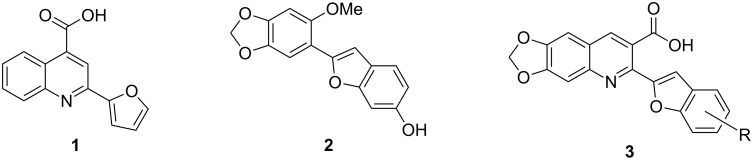
Structures of compounds **1**, **2** and **3**.

Heterocyclic systems containing a quinoline nucleus are an important group of compounds in medicinal chemistry, and are ubiquitous sub-structures associated with biologically active natural products [1–4]. Some quinoline compounds especially those containing heterocyclic systems at 2-position have been shown to display a wide spectrum of biological activities such as cytotoxic, anti-inflammatory and antifungal behavior [5–6]. For example, 2-(furan-2-yl)quinoline-4-carboxylic acid (and analogues) (**1**, Figure 1) was reported to inhibit *C. albicans* prolyl-tRNA synthetase and displayed potent in vitro antifungal activity against dermatophytes [6]. Consequently, studies concerning novel 2-heteroarylquinoline derivatives appear frequently in the literature [7–8].

In a nature’s collection of biologically active heterocycles, 2-heteroarylbenzofuran ring systems are widely distributed in nature and have been reported to have antiviral, antioxidant and antifungal activities [9–13]. For example, cicerfuran (**2**, Figure 1), obtained from the roots of a wild species of chickpea, *Cier bijugum*, was reported to be a major factor in the defense system against Fusarium wilt [12–13]. On the other hand, the methylenedioxy unit, present in many natural products such as safrole [14], Leucettamine B [15], and Steganacin [16], exhibits a wide variety of biological activities [17–20]. The methylenedioxy unit can be identified in the clinical antitumor agents etoposide, teniposide [21] and lignan lactone podophyllotoxin [22], and structure–activity relationships have shown that the methylenedioxy moiety is fundamental for cytotoxic activity since it can be metabolized by CYP to form metallo–carbene intermediates which may be responsible for the observed antitumor activity of lignans [23–24]. The presence of the methylenedioxy moiety in some other bioactive molecules drastically alters their pharmacological properties. For example, among the tested 2-phenylquinolin-4-ones (2-PQs) with potent cytotoxicity against human cancer cell lines, the methylenedioxy-bearing 2-PQ was identified as the most active compound in vivo [25].

In light of these findings and in view of structural diversity which plays a prominent role in medicinal and combinatorial chemistry and which leads to a faster and more efficient lead generation in new drug discovery [26], we felt that it would be of interest to construct new prototypes combining the methylenedioxy moiety, quinoline ring system and benzo[*b*]furan framework in the same molecule. Such compounds might be important for pharmacological studies or in the development of new medicinal products with interesting properties. Therefore, in continuation of our studies concerning the preparation of potential biologically active heterocyclic compounds [27–30], we now report herein a facile and inexpensive procedure for the preparation of novel hybrid molecules, i.e., 2-(benzofuran-2-yl)-6,7-methylenedioxyquinoline-3-carboxylic acid derivatives (**3**, Figure 1) under mild conditions.

## Results and Discussion

**Scheme 1:**
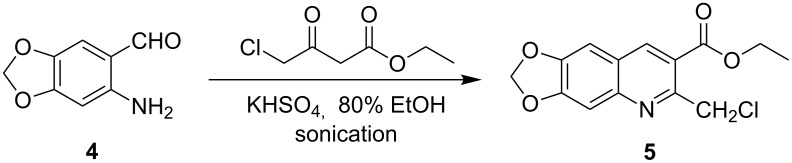
Synthesis of ethyl 2-chloromethyl-6,7-methylenedioxyquinoline-3-carboxylate (**5**).

The targeted 2-(benzofuran-2-yl)-6,7-methylenedioxyquinoline-3-carboxylic acid derivatives **3a**–**g** were synthesized via a two-step procedure, starting from ethyl 2-chloromethyl-6,7-methylenedioxyquinoline-3-carboxylate (**5**), which was obtained by the Friedländer condensation reaction of 2-amino-4,5-methylenedioxybenzaldehyde (**4**) with ethyl 4-chloro-3-oxobutanoate as outlined in Scheme 1. In this reaction, we found that the best results could be achieved when the reaction was carried out under ultrasound irradiation conditions at 80 °C using KHSO_4_ as catalyst in 80% EtOH as solvent. The resulting product **5** was obtained in good yield (74%) after purification by a flash chromatography on silica gel (eluent: petroleum ether/ethyl acetate = 5:1).

**Scheme 2:**
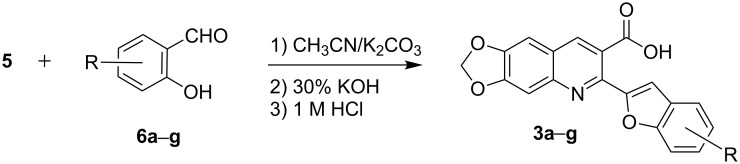
One-pot synthesis of the targeted compounds **3a**–**g**.

The next step involves the construction of the benzofuran moiety from intermediate **5**. For the construction of 2-substituted benzofurans, the most widely used approach involves the palladium-catalyzed heteroannulation of 2-halophenols with a terminal alkyne via a tandem Sonogashira coupling-5-endo-dig-cyclization, largely based on the methods of Larock and his co-workers [31–34]. Recently, others less popular approaches for the synthesis of 2-substituted benzofurans have included *p*-toluenesulfonic acid-mediated cyclization of *o*-(1-alkynyl)anisoles to obtain 2-arylsubstituted benzofurans [35], rearrangement and cyclization reactions of 2-hydroxybenzophenones with Corey–Chaykovsky reagent [36], cyclization of 2-acyloxy-1-bromomethylarenes with Cr(II)Cl_2_/BF_3_·OEt_2_ catalyst [37], and boron tribromide-promoted tandem deprotection–cyclization of 2-methoxyphenylacetones [38], 2-methoxyphenylmethanols [39] and 2-hydroxy-3-arylpropenoic acids to yield 2-methyl, 2-carboxy, and 2-arylbenzo[*b*]furans, respectively [40]. However, these methods often require expensive catalysts and/or multi-step syntheses. Herein, we demonstrate an attractive one-pot procedure to afford the desired 2-(benzofuran-2-yl)-6,7-methylenedioxyquinoline-3-carboxylic acid derivatives (**3a**–**g**) by the reaction of **5** with substituted salicylaldehydes **6a**–**g**. The reaction proceeds via in situ Williamson ether formation followed by ester hydrolysis and intramolecular cyclization (Scheme 2). The procedure uses an inexpensive inorganic base as catalyst under mild reaction conditions and gives straightforward and easy access for the incorporation of benzofuran core onto the quinoline nucleus at 2-position, to give the desired compounds in with good yields via a simple workup.

In this one-pot method, the 2-chloromethylquinoline **5** was first subjected to the Williamson reaction with various salicylaldehydes **6a**–**g** in the presence of K_2_CO_3_ as catalyst in refluxing CH_3_CN. Acetonitrile was employed in our method because of its low boiling point and leads to a much more convenient workup procedure. Thus, once the Williamson reaction was complete as observed on TLC, we simply evaporated CH_3_CN to dryness, added 30% ethanolic potassium hydroxide solution (10 mL) to the residue and heated the mixture under reflux for 4 hours. Thus, the targeted products **3a**–**g** were obtained in 52–82% overall yields. Encouraged by these results, we also attempted the reaction of **5** with 2-hydroxy-1-naphthaldehyde (**6h**) under similar reaction conditions with the aim of constructing a novel naphthofuran derivative. Interestingly, 2-hydroxy-1-napthaldehyde was equally amenable to the conditions and gave the corresponding 2-(naphtho[2,1-*b*]furan-2-yl)-6,7-methylenedioxyquinoline-3-carboxylic acid (**3h**) in good yield (69%). The yields and melting points of all the synthesized products **3a**–**h** are listed in Table 1. It is noteworthy that in the one-pot reaction the use of 30% ethanolic potassium hydroxide solution is sufficient to promote the reaction and there were no improvement in the reaction rates and yields by increasing the amount of potassium hydroxide or by using other bases. Compounds **3a**–**h** are novel and their structures were established based on spectral data and elemental analyses. For example, the IR spectrum of **3a** exhibited the presence of hydroxyl and carbonyl groups of carboxyl moiety at 3449 and 1697 cm^−1^, respectively. Its structure was unequivocal proven by the ^1^H NMR spectrum. Its ^1^H NMR spectrum showed no signals attributable to chloromethyl and ester groups but contained a broad singlet at 13.42 ppm for carboxylic proton. Particularly characteristic was the presence of the furan proton singlet at 7.52 ppm along with signals for seven aromatic ring protons between 7.37–8.57 ppm, which is consistent with the attachment of the nascent benzofuran ring moiety to the quinoline substrate. In addition, the structure of **3a** was further confirmed by its ^13^C NMR spectrum, which revealed the presence of carboxyl carbon and methylenedioxy carbon at 168.12 and 102.68 ppm, respectively, along with the signals due to the aromatic carbons. The mass spectrum of **3a** contained a quasi-molecular ion peak at *m/z* 334.1 ([M + H]^+^) which was also indicative of the proposed structure. The other synthesized compounds exhibited similar spectral characteristics. Elemental analysis was in agreement with the theoretical values.

**Table 1:** Synthesis of 2-benzofuranyl-6,7-methylenedioxyquinoline-3-carboxylic acids **3a**–**h**.

Entry	Aldehyde **6**		Product **3**		Yield (%)^a^	Mp (°C)

1	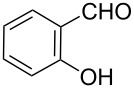	**6a**	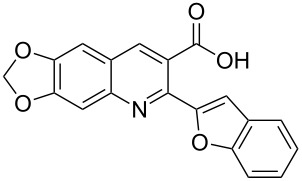	**3a**	74	201–202
2	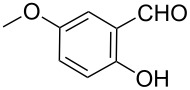	**6b**	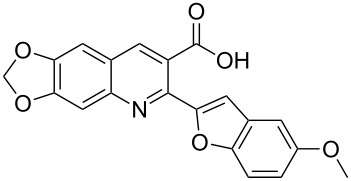	**3b**	76	241–242
3	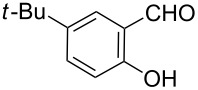	**6c**	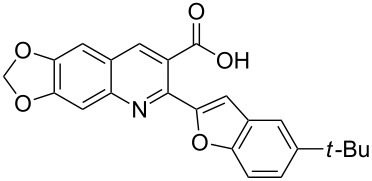	**3c**	82	260–261
4	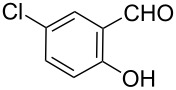	**6d**	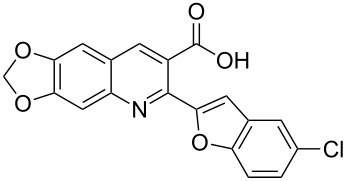	**3d**	80	233–234
5	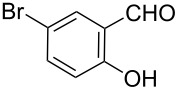	**6e**	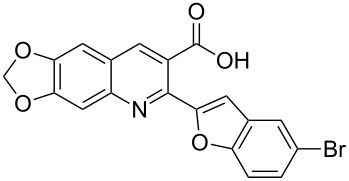	**3e**	73	192–193
6	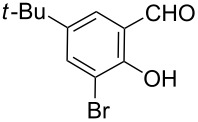	**6f**	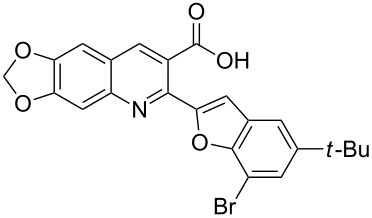	**3f**	64	239–240
7	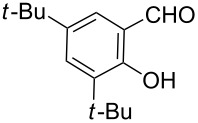	**6g**	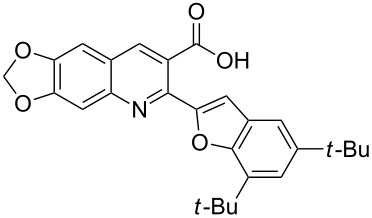	**3g**	52	291–292
8	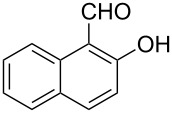	**6h**	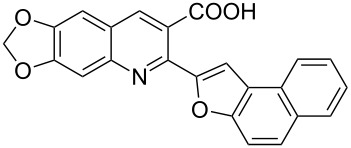	**3h**	69	232–233

^a^Isolated yield.

As shown in Table 1, with the exception of **3g** (entry 7), a series of 2-(benzofuran-2-yl)quinoline-3-carboxylic acid derivatives **3** were readily prepared under mild reaction conditions in good to high yield from the one-pot reaction of 2-chloromethylquinoline **5** with a variety of salicylaldehydes. In the case of entry 7, the reaction of 3,5-di-*tert*-butylsalicylaldedyde (**6g**) with **5** gave the corresponding product **3g** in a moderate yield of 52%, which might be attributed to the sterically hindered nature of the bulky *tert*-butyl group at 3-position of **6g.** The ease of isolation of all the products is notable; after acidification with 1 M HCl the products were isolated as the major reaction products. The method failed when strong electron-withdrawing groups such as nitro or cyano groups were present on the salicylaldehyde. These reactions were found to be very complex and we could not separate and obtain any of the desired products in appreciable yields in these cases. Instead, intractable complex mixtures were observed on TLC.

**Scheme 3:**
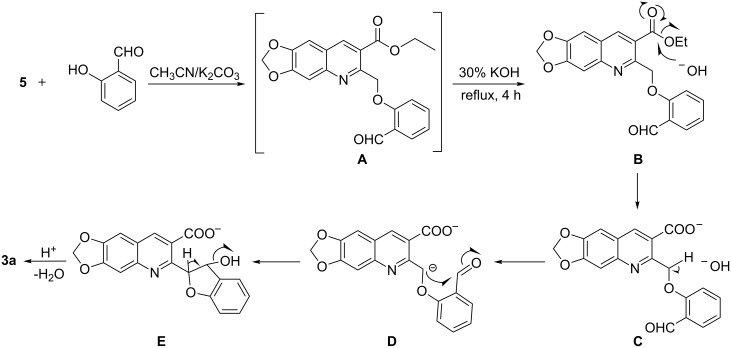
Possible mechanistic pathway of formation of 2-(benzofuran-2-yl)-6,7-methylenedioxyquinoline-3-carboxylic acid (**3a**).

To illustrate the reaction mechanism, the formation of **3a** is outlined in Scheme 3. The in situ base-mediated ester hydrolysis of the initially formed Williamson product **A** resulted in the abstraction of acidic proton from the active methylene group in **C** and subsequent attack of the carbanion at the aldehyde carbonyl carbon with the formation of the five-membered cyclic system **E**. This is then followed by dehydration to yield **3a**. The proposed mechanism is very similar to the formation of naphthofurans via intramolecular condensation in the presence of triethylamine reported by Srivastava et al [41].

## Conclusion

In summary, the present method offers a straightforward and facile synthetic route for the preparation of a variety of 2-benzofuranyl-6,7-methylenedioxyquinoline-3-carboxylic acids **3a**–**h**. Ready availability of starting materials, mild reaction conditions, experimental simplicity and satisfactory yields contribute to the usefulness of this method. Biological activity of the synthesized compounds remains to be studied.

## Experimental

Melting points (uncorrected) were determined with a WRS-1B melting point apparatus and are uncorrected. Ultrasonication was performed in a KQ-250B medical ultrasound cleaner with a frequency of 40 KHz and output power of 250W (built in heating 30–80 °C thermostatically adjustable). ^1^H NMR and ^13^C NMR spectra were recorded on a Bruker Avance NMR spectrometer using CDCl_3_ or DMSO-*d*_6_ as the solvent. The reported chemical shifts (δ values) are given in parts per million downfield from tetramethylsilane (TMS) as the internal standard. The mass spectra were determined using a MSD VL ESI1 spectrometer. Elemental analyses was performed for C, H, and N using an Elementar Vario EL-III element analyzer and were within ± 0.4% of the calculated values. The progress of reactions was monitored by thin layer chromatography (TLC) on silica gel GF254 using ethyl acetate as mobile phase.

### Preparation of ethyl 2-chloromethyl-6,7-methylenedioxyquinoline-3-carboxylate (**5**)

To a stirred solution of 2-amino-4,5-methylenedioxybenzaldehyde (**4**) (4.73 g, 29 mmol) and ethyl 4-chloro-3-oxobutanoate (8.11 g, 49 mmol) in 80% ethanol (160 mL), was added a solution of potassium hydrogen sulfate (2.27 g, 17 mmol) in water (20 mL). The resulting mixture was sonicated at 80 °C for 3 h. After the reaction was complete (TLC), the mixture was cooled to room temperature, poured into water and filtered to give the crude product, which was then purified by silica gel column chromatography with ethyl acetate/petroleum ether (5:1) as eluent. The title compound was obtained as a yellow solid in 74% yield, mp 199–203 °C, IR (KBr) ν/cm^−1^: 1710, 1251, 1231, 948; ^1^H NMR (CDCl_3_, 300 MHz): δ (ppm) 1.48 (t, *J* = 7.1 Hz, 3H, CH_3_), 4.48 (q, *J* = 7.1 Hz, 2H, CH_2_), 5.22 (s, 2H, ArCH_2_Cl), 6.17 (s, 2H, OCH_2_O), 7.13 (s, 1H, quinoline-H), 7.40 (s, 1H, quinoline-H), 8.62 (s, 1H, quinoline-H); MS (ESI, *m*/*z*): 294.0 [M + H]^+^; Anal. Calcd for C_14_H_12_ClNO_4_: C, 57.25; H, 4.12; N, 4.77. Found: C, 57.07; H, 4.29; N, 4.70.

### General procedure for the synthesis of 2-(benzofuran-2-yl)-6,7-methylenedioxyquinoline-3-carboxylic acids **3a**–**h**

A mixture of ethyl 2-chloromethyl-6,7-methylenedioxyquinoline-3-carboxylate (**5**) (0.5 mmol, 0.147 g), the corresponding salicylaldehyde or 2-hydroxy-1-naphthaldehyde (0.5 mmol) and anhydrous K_2_CO_3_ (2.5 mmol, 0.400 g) was stirred in refluxing CH_3_CN (12 mL). The conversion was monitored by TLC. After completion, CH_3_CN was evaporated to dryness. Then 30% ethanolic potassium hydroxide solution (15 mL) was added to the residue and the mixture heated under reflux for 4 h, cooled, and acidified with 1 M hydrochloric acid solution. The resulting crude product was recrystalized from ethyl acetate to afford **3a**–**h**. The melting points and yields of all the compounds are summarized in Table 1 and the spectral and analytical data are given below.

#### 2-(Benzofuran-2-yl)-6,7-methylenedioxyquinoline-3-carboxylic acid (**3a**)

The compound was obtained as a yellow solid. IR (KBr) ν/cm^−1^: 3449, 1697, 1258, 1110; ^1^H NMR (DMSO-*d*_6_, 600 MHz): δ (ppm) 6.32 (s, 2H, OCH_2_O), 7.37 (t, *J* = 7.2 Hz, 1H, benzofuran-H), 7.44 (t, *J* = 7.2 Hz, 1H, benzofuran-H), 7.50 (s, 1H, quinoline-H), 7.52 (s, 1H, furan-H), 7.56 (s, 1H, quinoline-H), 7.68 (d, *J* = 8.4 Hz, 1H, benzofuran-H), 7.82 (d, *J* = 7.8 Hz, 1H, benzofuran-H), 8.57 (s, 1H, quinoline-H), 13.42 (s, 1H, OH); ^13^C NMR (DMSO-*d*_6_, 150 MHz): δ (ppm) 102.68, 103.11, 104.82, 106.49, 111.38, 121.90, 123.35, 123.65, 125.32, 128.32, 135.97, 143.69, 146.06, 148.80, 152.42, 154.53, 154.63, 168.12; MS (ESI, *m*/*z*): 334.1 [M + H]^+^; Anal. Calcd for C_19_H_11_NO_5_: C, 68.47; H, 3.33; N, 4.20. Found: C, 68.33; H, 3.57; N, 4.12.

#### 2-(5-Methoxybenzofuran-2-yl)-6,7-methylenedioxyquinoline-3-carboxylic acid (**3b**)

The compound was obtained as a yellow solid. IR (KBr) ν/cm^−1^: 3439, 1618, 1236, 1037; ^1^H NMR (DMSO-*d*_6,_ 600 MHz): δ (ppm) 3.82 (s, 3H, OMe), 6.29 (s, 2H, OCH_2_O), 6.97 (dd, *J* = 8.4, 2.4 Hz, 1H, benzofuran-H), 7.26 (d, *J* = 2.4 Hz, 1H, benzofuran-H), 7.36 (s, 1H, quinoline-H), 7.46 (s, 1H, quinoline-H), 7.50–7.52 (m, 2H, furan-H and benzofuran-H), 8.53 (s, 1H, quinoline-H), 13.36 (s, 1H, OH); ^13^C NMR (DMSO-*d*_6_, 150 MHz): δ (ppm) 55.68, 102.70, 103.13, 103.86, 104.80, 106.64, 111.93, 114.26, 123.55, 124.38, 128.91, 136.10, 143.69, 146.15, 148.80, 149.66, 152.51, 155.17, 155.93, 168.76; MS (ESI, *m*/*z*): 364.1 [M + H]^+^; Anal. Calcd for C_20_H_13_NO_6_: C, 66.12; H, 3.61; N, 3.86. Found: C, 66.06; H, 3.80; N, 3.69.

#### 2-(5-*tert*-Butylbenzofuran-2-yl)-6,7-methylenedioxyquinoline-3-carboxylic acid (**3c**)

The compound was obtained as a yellow solid. IR (KBr) ν/cm^−1^: 3470, 1745, 1253, 1116; ^1^H NMR (DMSO-*d*_6_, 600 MHz) δ (ppm): 1.37 (s, 9H, *tert*-butyl), 6.29 (s, 2H, OCH_2_O), 7.38 (s, 1H, quinoline-H), 7.44 (dd, *J* = 9.0, 1.2 Hz, 1H, benzofuran-H), 7.46 (s, 1H, quinoline-H), 7.51 (s, 1H, furan-H), 7.53 (d, *J* = 9.0 Hz, 1H, benzofuran-H), 7.73 (d, *J* = 1.2 Hz, 1H, benzofuran-H), 8.53 (s, 1H, quinoline-H), 13.36 (s, 1H, OH); ^13^C NMR (DMSO-*d*_6_, 150 MHz): δ (ppm) 31.69, 34.59, 102.69, 103.13, 104.81, 106.66, 110.69, 117.84, 123.26, 123.54, 128.01, 136.10, 143.83, 145.93, 146.15, 148.77, 152.49, 152.95, 154.59, 168.80; MS (ESI, *m*/*z*): 390.1 [M + H]^+^; Anal. Calcd for C_23_H_19_NO_5_: C, 70.94; H, 4.92; N, 3.60. Found: C, 70.85; H, 5.07; N, 3.57.

#### 2-(5-Chlorobenzofuran-2-yl)-6,7-methylenedioxyquinoline-3-carboxylic acid (**3d**)

The compound was obtained as a yellow solid. IR (KBr) ν/cm^−1^: 3445, 1696, 1247, 1119; ^1^H NMR (DMSO-*d*_6_, 600 MHz): δ (ppm) 6.30 (s, 2H, OCH_2_O), 7.40 (s, 1H, quinoline-H), 7.41 (dd, *J* = 9.0, 1.8 Hz, 1H, benzofuran-H), 7.47 (s, 1H, quinoline-H), 7.53 (s, 1H, furan-H), 7.66 (d, *J* = 9.0 Hz, 1H, benzofuran-H), 7.84 (d, *J* = 1.8 Hz, 1H, benzofuran-H), 8.60 (s, 1H, quinoline-H), 13.42 (s, 1H, OH); ^13^C NMR (DMSO-*d*_6_, 150 MHz): δ (ppm) 102.79, 103.16, 104.84, 106.07, 112.98, 121.28, 123.83, 124.38, 125.21, 127.73, 129.92, 136.42, 143.41, 146.23, 149.02, 152.67, 153.11, 156.10, 168.46; MS (ESI, *m*/*z*): 368.0 [M + H]^+^; Anal. Calcd for C_19_H_10_ClNO_5_: C, 62.06; H, 2.74; N, 3.81. Found: C, 61.98; H, 2.83; N, 3.77.

#### 2-(5-Bromobenzofuran-2-yl)-6,7-methylenedioxyquinoline-3-carboxylic acid (**3e**)

The compound was obtained as a yellow solid. IR (KBr) ν/cm^−1^: 3442, 1693, 1247, 1121; ^1^H NMR (DMSO-*d*_6_, 600 MHz): δ (ppm) 6.35 (s, 2H, OCH_2_O), 7.46 (s, 1H, quinoline-H), 7.53 (s, 1H, furan-H), 7.57 (d, *J* = 9.0 Hz, 1H, benzofuran-H), 7.58 (s, 1H, quinoline-H), 7.67 (d, *J* = 8.4 Hz, 1H, benzofuran-H), 8.04 (s, 1H, benzofuran-H), 8.65 (s, 1H, quinoline-H), 13.49 (s, 1H, OH); ^13^C NMR (DMSO-*d*_6_, 150 MHz): δ (ppm) 102.78, 103.16, 104.84, 105.92, 113.44, 115.62, 123.83, 124.30, 127.86, 130.56, 136.43, 143.38, 146.24, 149.02, 152.66, 153.44, 155.90, 168.46; MS (ESI, *m*/*z*): 412.0 [M + H]^+^; Anal. Calcd for C_19_H_10_BrNO_5_: C, 55.36, H, 2.45, N, 3.40. Found: C, 55.27, H, 2.69, N, 3.33.

#### 2-(7-Bromo-5-*tert*-butylbenzofuran-2-yl)-6,7-methylenedioxyquinoline-7-carboxylic acid (**3f**)

The compound was obtained as a yellow solid. IR (KBr) ν/cm^−1^: 3445, 1741, 1242, 1112; ^1^H NMR (DMSO-*d*_6_, 600 MHz): δ (ppm) 1.36 (s, 9H, *tert*-butyl), 6.30 (s, 2H, OCH_2_O), 7.46 (s, 1H, quinoline-H), 7.48 (s, 1H, benzofuran-H), 7.53 (s, 1H, furan-H), 7.61 (s, 1H, quinoline-H), 7.74 (s, 1H, benzofuran-H), 8.60 (s, 1H, quinoline-H), 13.38 (s, 1H, OH); ^13^C NMR (DMSO-*d*_6_, 150 MHz): δ (ppm) 31.51, 34.79, 102.70, 102.77, 103.19, 104.85, 107.16, 117.59, 123.85, 124.56, 125.72, 129.42, 136.54, 143.64, 146.22, 148.11, 148.97, 149.92, 152.65, 155.48, 168.30; MS (ESI, *m*/*z*): 468.0 [M + H]^+^; Anal. Calcd for C_23_H_18_BrNO_5_: C, 58.99; H, 3.87; N, 2.99. Found: C 58.75; H, 3.96; N 2.69.

#### 2-(5,7-Di-*tert*-butylbenzofuran-2-yl)-6,7-methylenedioxyquinoline-3-carboxylic acid (**3g**)

The compound was obtained as a yellow solid. IR (KBr) ν/cm^−1^: 3457, 1710, 1251, 1113; ^1^H NMR (DMSO-*d*_6_, 600 MHz): δ (ppm) 1.37 (s, 9H, *tert*-butyl), 1.43 (s, 9H, *tert*-butyl), 6.29 (s, 2H, OCH_2_O), 7.26 (d, *J* = 1.2 Hz, 1H, benzofuran-H), 7.41 (s, 1H, quinoline-H), 7.44 (s, 1H, quinoline-H), 7.51 (s, 1H, furan-H), 7.56 (d, *J* = 1.2 Hz, 1H, benzofuran-H), 8.54 (s, 1H, quinoline-H), 13.27 (s, 1H, OH); ^13^C NMR (150 MHz, DMSO-*d*_6_): δ (ppm) 29.97, 31.75, 34.08, 34.67, 102.67, 103.13, 104.75, 106.25, 115.73, 119.39, 123.53, 128.57, 133.71, 136.14, 144.05, 145.55, 146.16, 148.69, 151.37, 152.46, 154.34, 168.53; MS (ESI, *m*/*z*): 446.2 [M + H]^+^; Anal. Calcd for C_27_H_27_NO_5_: C, 72.79; H, 6.11; N, 3.14. Found: C, 72.71; H, 6.26; N, 3.11.

#### 2-(Naphtho[2,1-*b*]furan-2-yl)-6,7-methylenedioxyquinoline-3-carboxylic acid (**3h**)

The compound was obtained as an orange solid. IR (KBr) ν/cm^−1^: 3433, 1601, 1258, 1034; ^1^H NMR (DMSO-*d*_6_, 600 MHz): δ (ppm) 6.30 (s, 2H, OCH_2_O), 7.47 (s, 1H, quinoline-H), 7.53 (s, 1H, furan-H), 7.57 (t, *J* = 7.8 Hz, 1H, naphth**-**H), 7.68 (t, *J* = 7.8 Hz, 1H, naphth-H), 7.81 (d, *J* = 9.0 Hz, 1H, naphth-H), 7.92 (d, *J* = 9.0 Hz, 1H, naphth-H), 8.08 (d, *J* = 8.4 Hz, 1H, naphth-H), 8.11 (s, 1H, quinoline-H), 8.44 (d, *J* = 8.4 Hz, 1H, naphth-H), 8.58 (s, 1H, quinoline-H), 13.39 (s, 1H, OH); ^13^C NMR (DMSO-*d*_6_, 150 MHz): δ (ppm) 90.29, 102.72, 103.21, 104.72, 105.93, 112.42, 123.51, 123.73, 123.92, 125.11, 126.36, 126.90, 127.49, 128.82, 130.13, 136.29, 143.87, 146.26, 148.76, 150.23, 152.57, 154.12, 155.35, 168.82; MS (ESI, *m*/*z*): 384.1 [M + H]^+^; Anal. Calcd for C_23_H_13_NO_5_: C, 72.06; H, 3.42; N, 3.65. Found: C, 71.96; H, 3.69; N, 3.51.

## Supporting Information

Supporting Information features ^1^H NMR and ^13^C NMR spectra of the substrate 2-chloromethyl-6,7-methylenedioxyquinoline-3-carboxylate (**5**) and 2-(benzofuran-2-yl)-6,7-methylenedioxyquinoline-3-carboxylic acids **3a**–**h**.

File 1 ^1^H NMR and ^13^C NMR spectra of the title compounds **5** and **3a**–**h**.
