# Supplementary material for: First synthesis of 2-(benzofuran-2-yl)-6,7-methylene dioxyquinoline-3-carboxylic acid derivatives
Source: Beilstein J Org Chem. 2011 Feb 15;7:210–7. doi: 10.3762/bjoc.7.28 (PMC3062989; doi:10.3762/bjoc.7.28)
Supplement: File 1 — 1H NMR and 13C NMR spectra of the title compounds 5 and 3a–h. [file Beilstein_J_Org_Chem-07-210-s001.pdf]

Supporting Information

for

First synthesis of 2-(benzofuran-2-yl)-6,7-methylene dioxyquinoline-3-carboxylic acid derivatives

Wentao Gao*, Jia Liu, Yun Jiang and Yang Li

Address: Institute of Superfine Chemicals, Bohai University, Jinzhou 121000, China

Email: Wentao Gao - bhuzh@163.com

* Corresponding author

^{1}H NMR and ^{13}C NMR spectra of the title compounds 5 and 3a–h

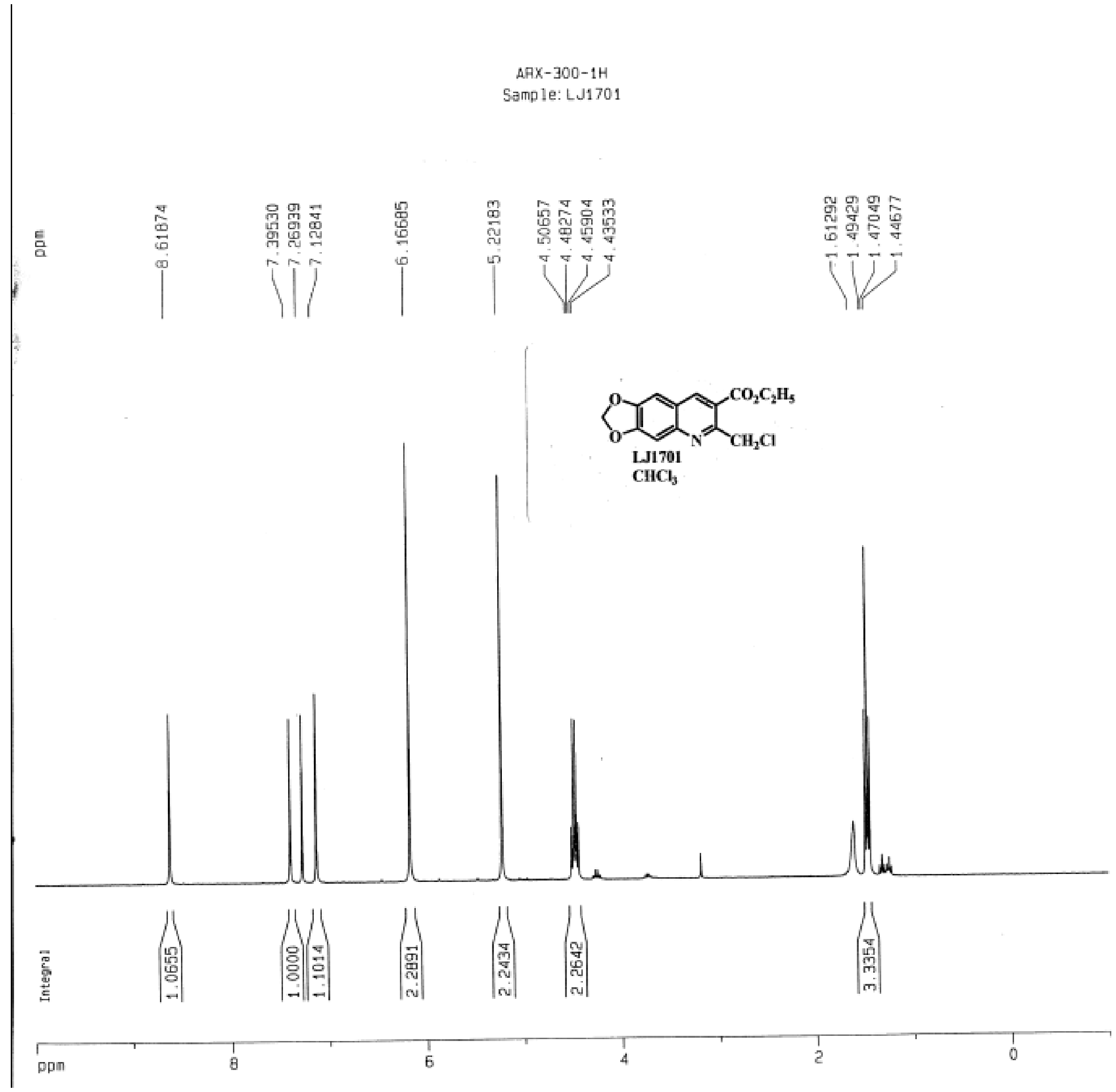

Figure S1: ^{1}H NMR spectrum of compound **5**.

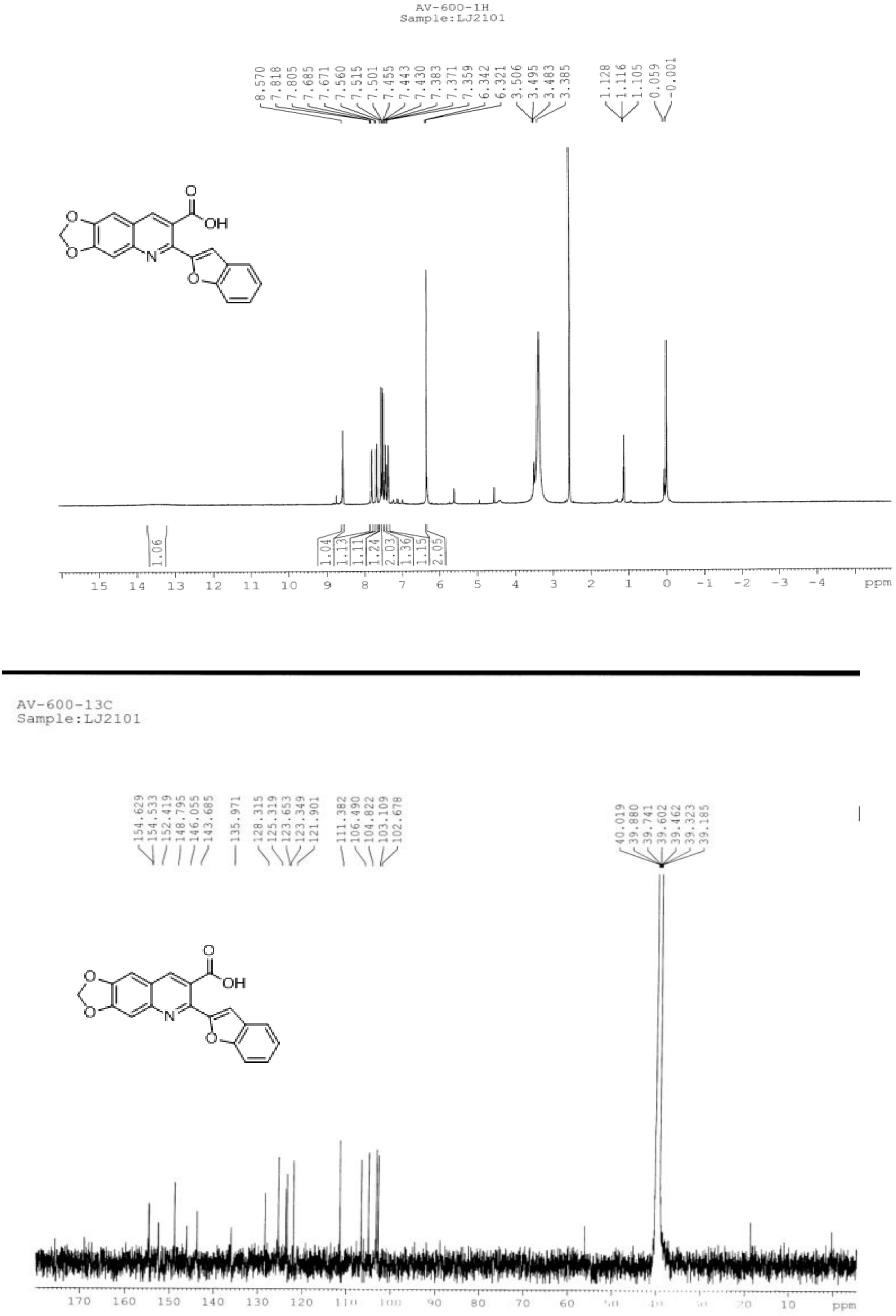

Figure S2: ^{1}H NMR and ^{13}C NMR spectra of **3a**.

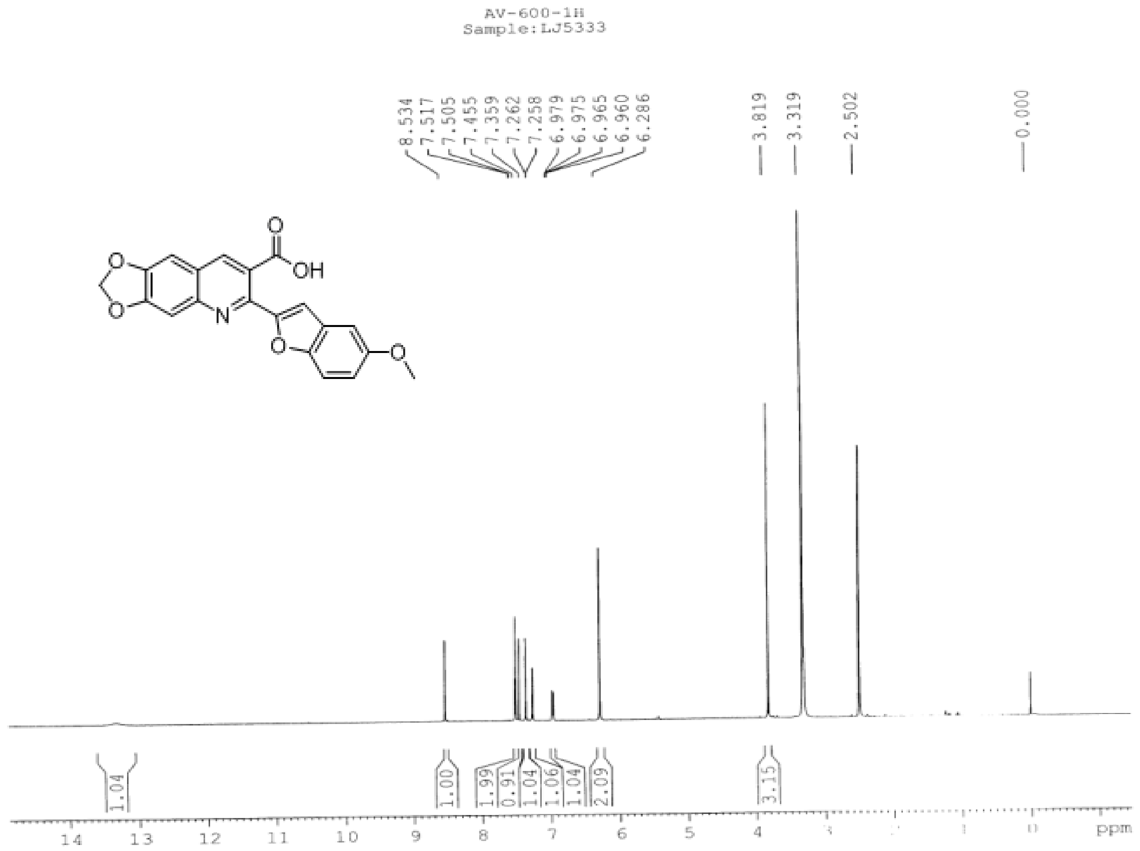

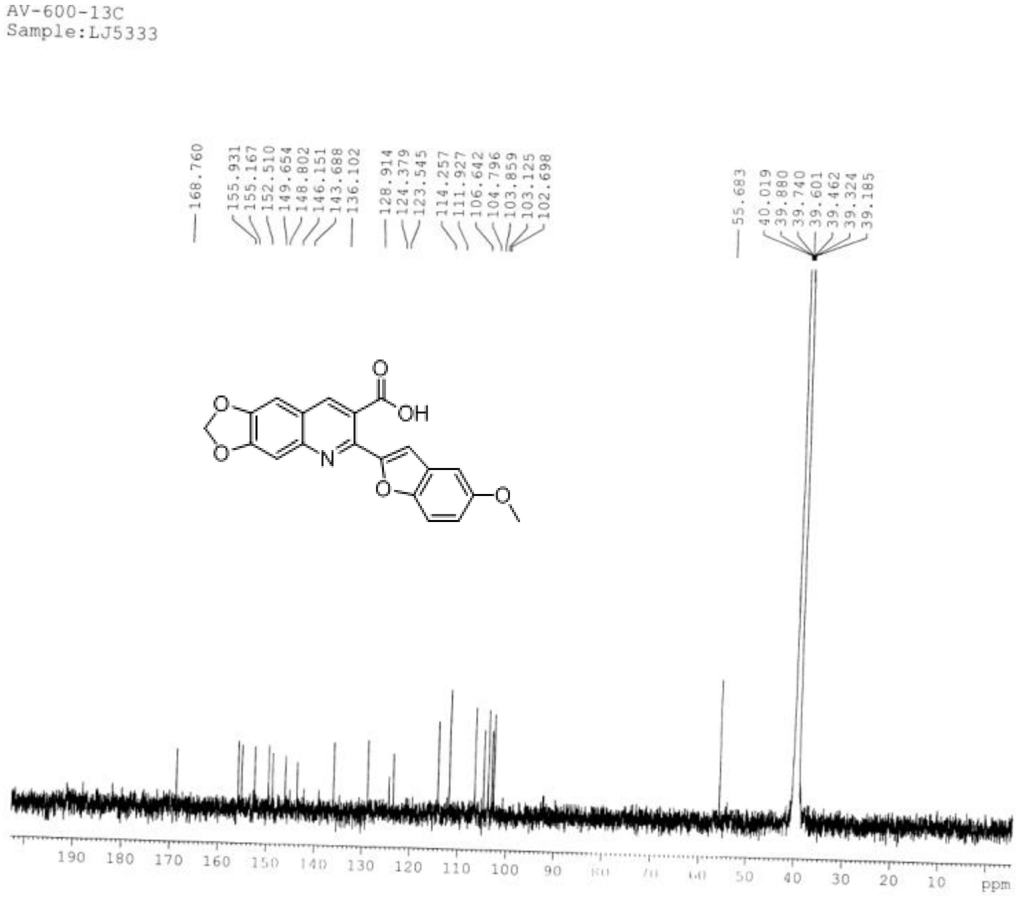

Figure S3: ^{1}H NMR and ^{13}C NMR spectra of **3b**.

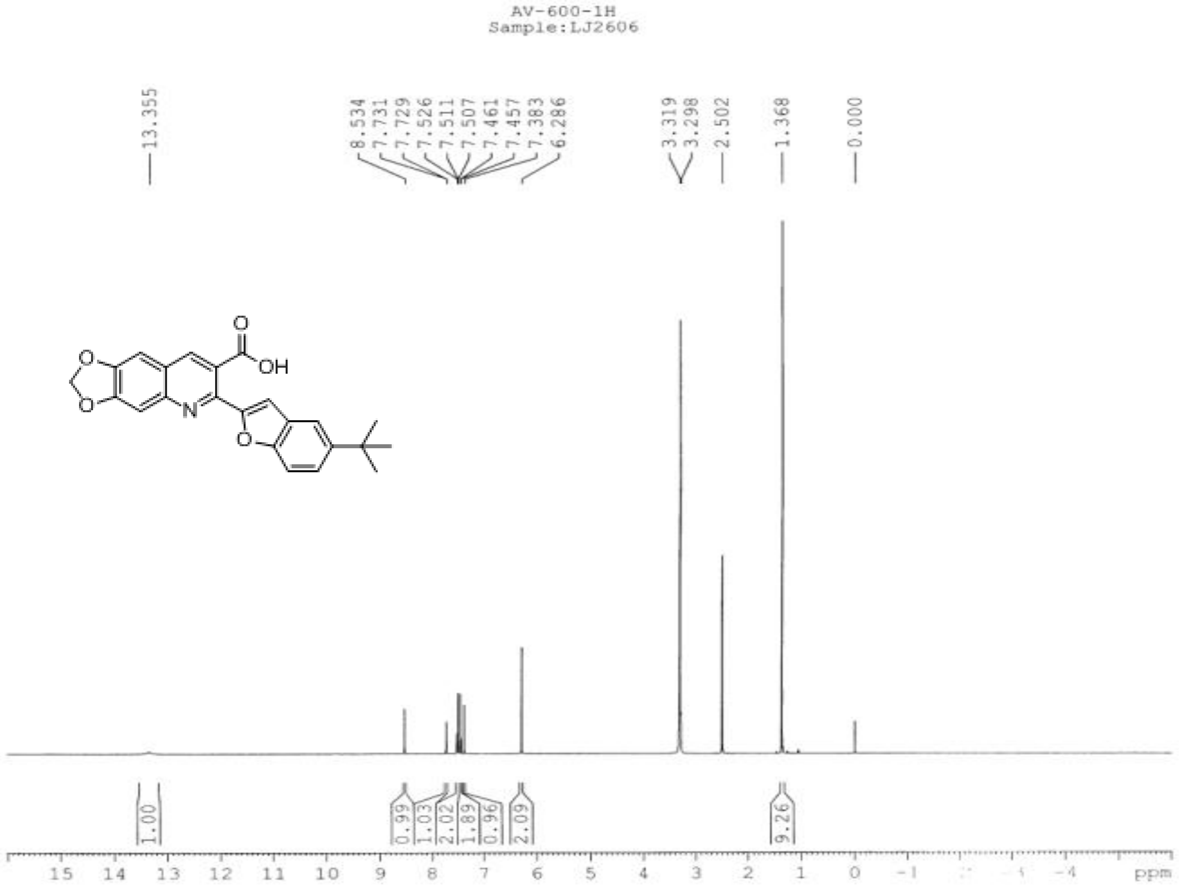

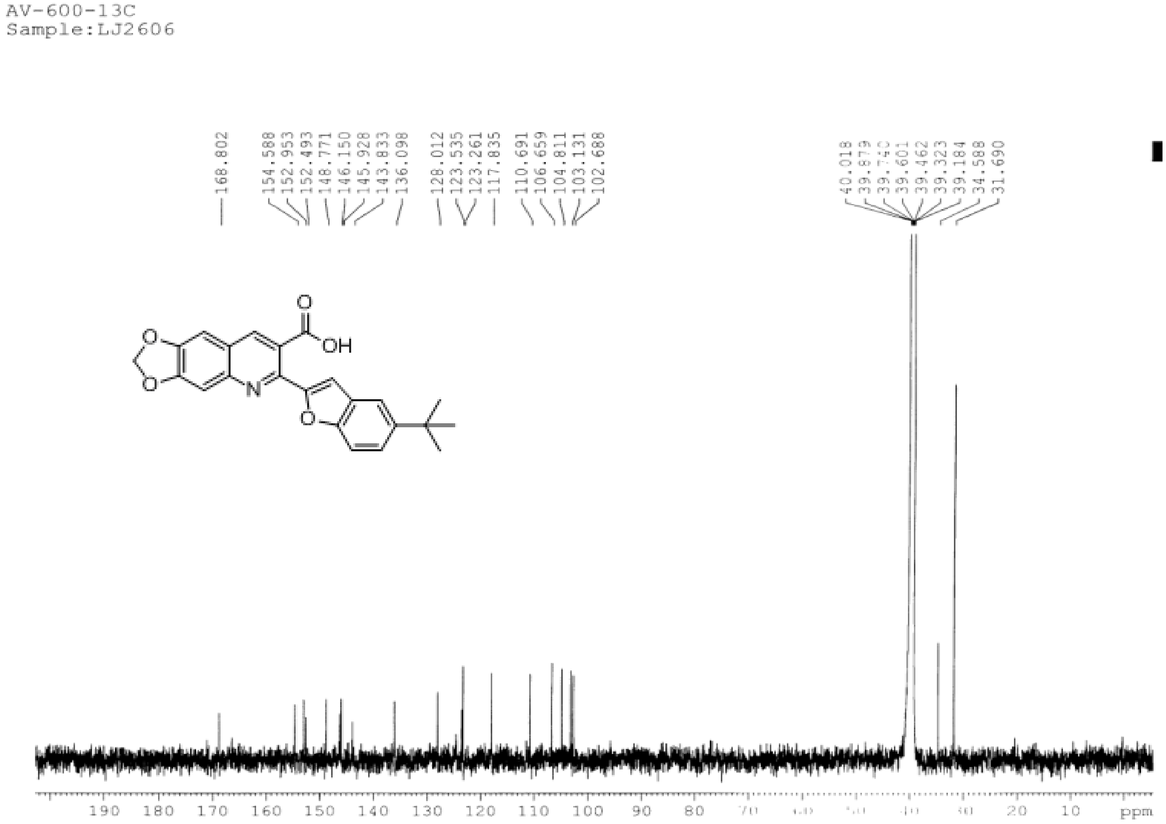

Figure S4: ^{1}H NMR and ^{13}C NMR spectra of **3c**.

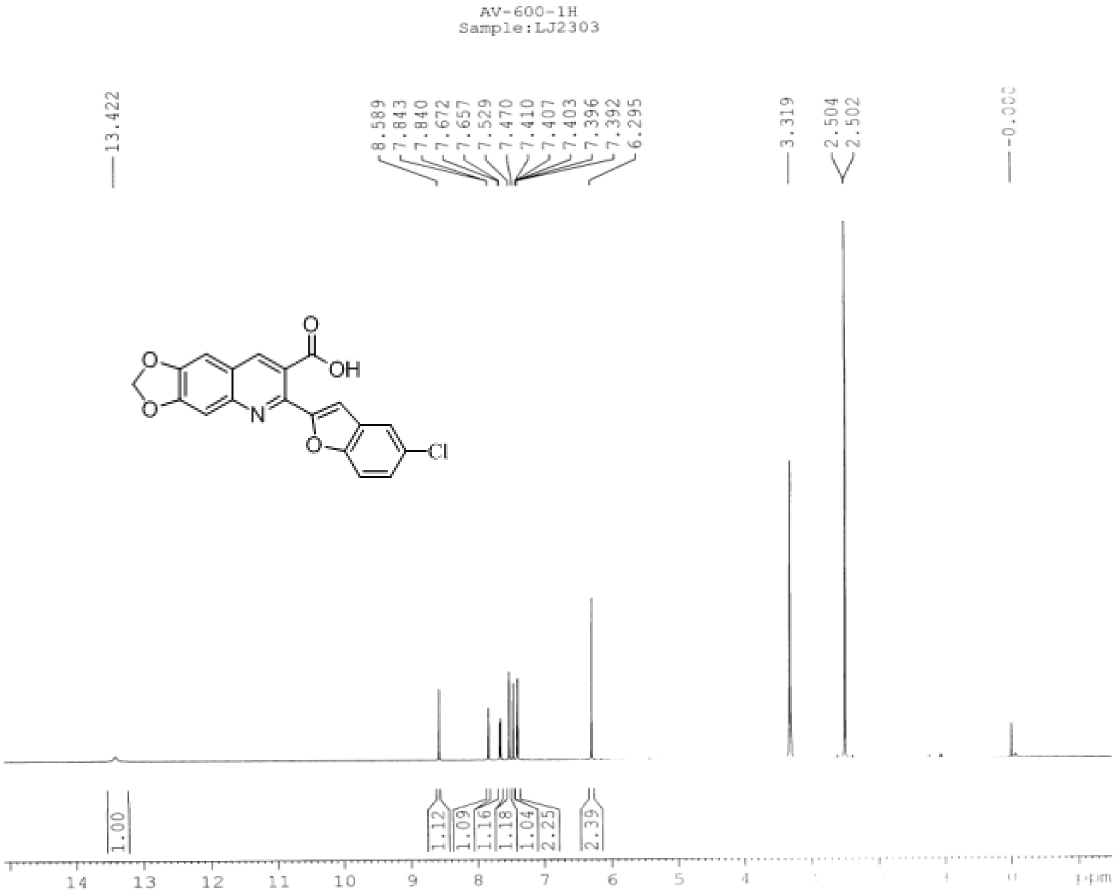

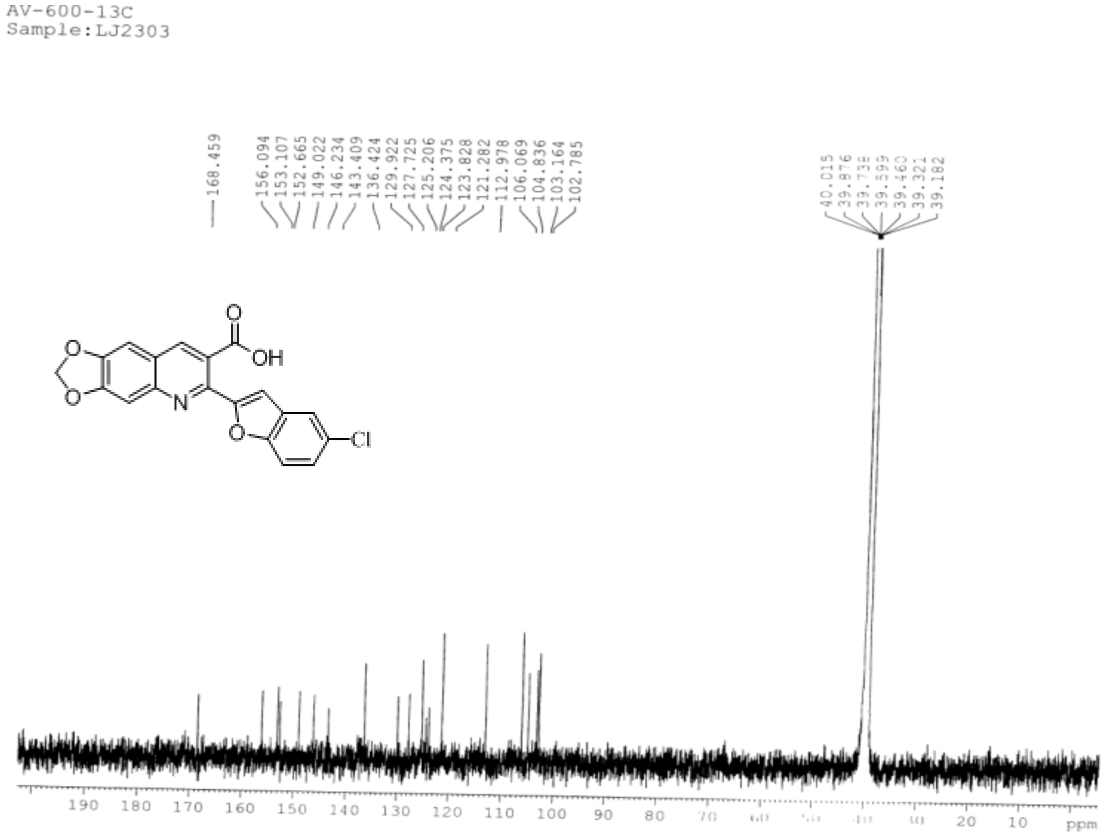

Figure S5: ^{1}H NMR and ^{13}C NMR spectra of **3d**.

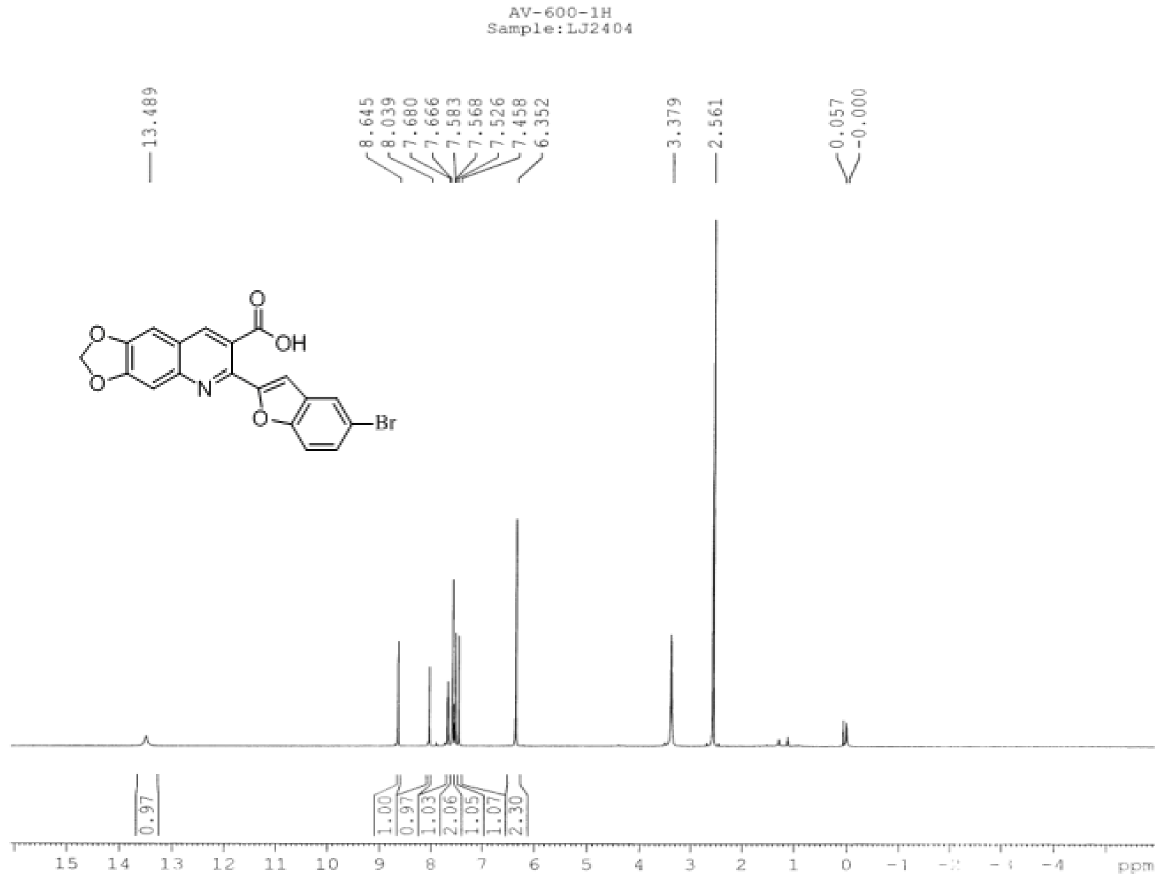

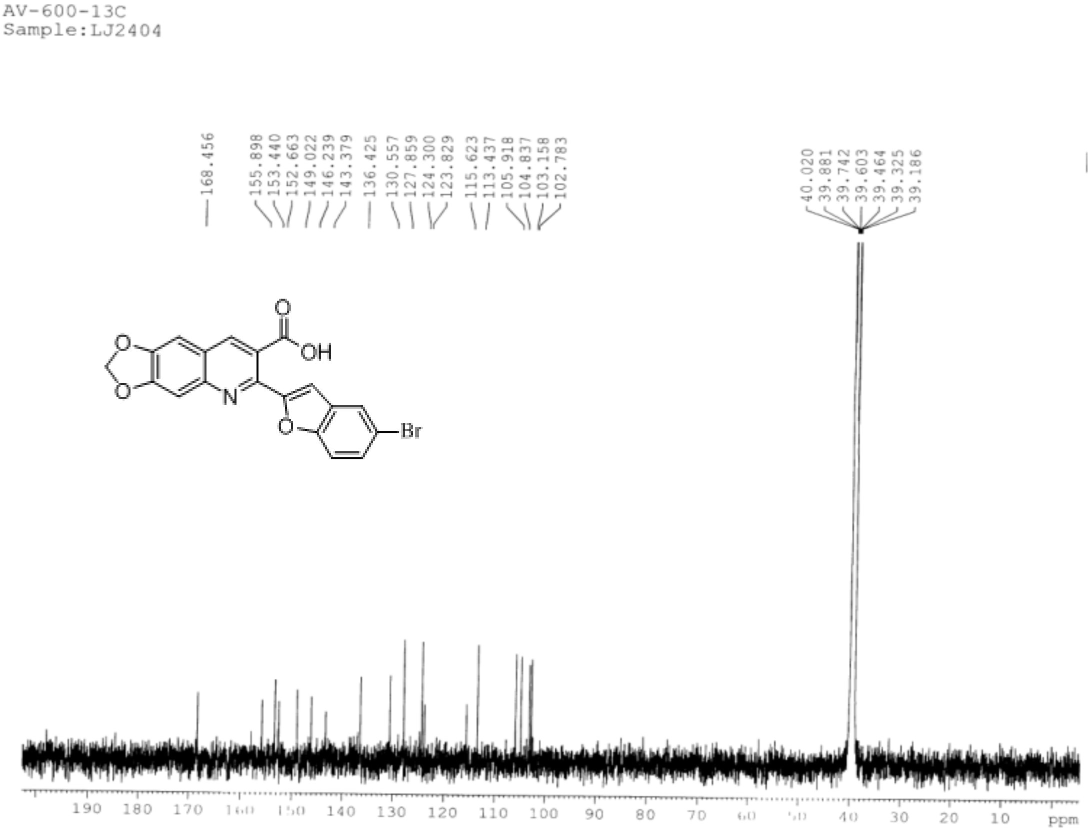

Figure S6: ^{1}H NMR and ^{13}C NMR spectra of **3e**.

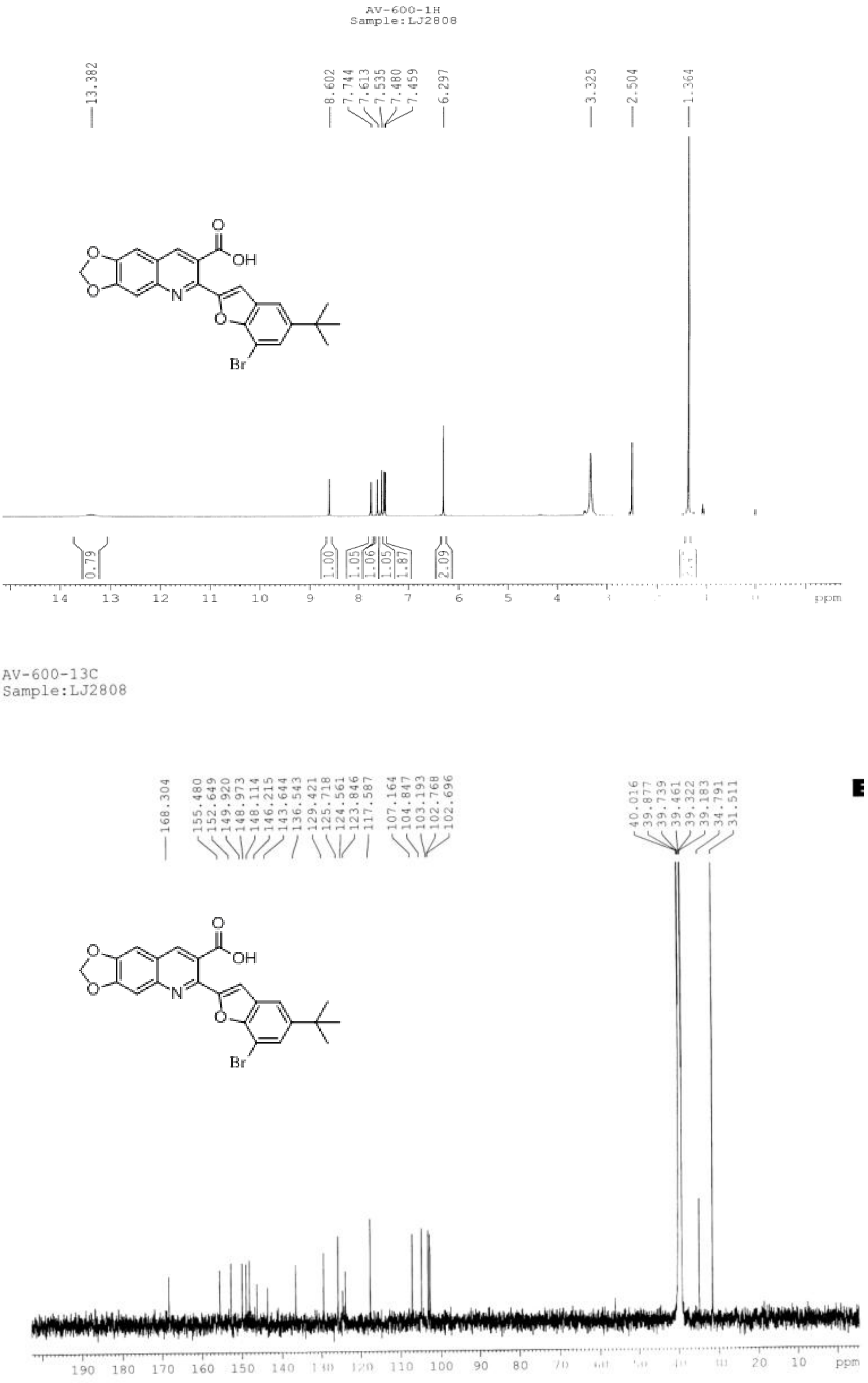

Figure S7: ^{1}H NMR and ^{13}C NMR spectra of **3f**.

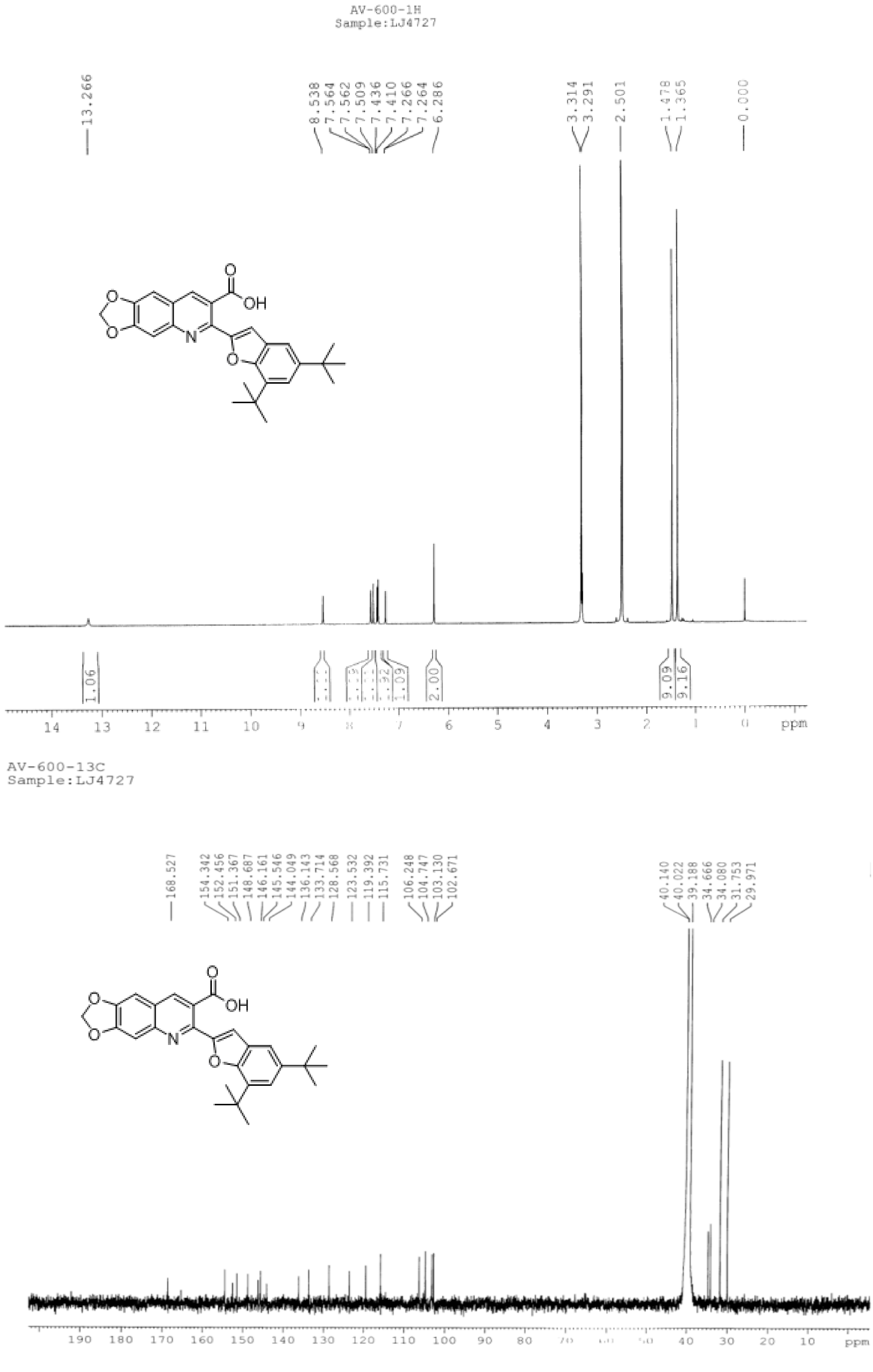

Figure S8: ^{1}H NMR and ^{13}C NMR spectra of **3g**.

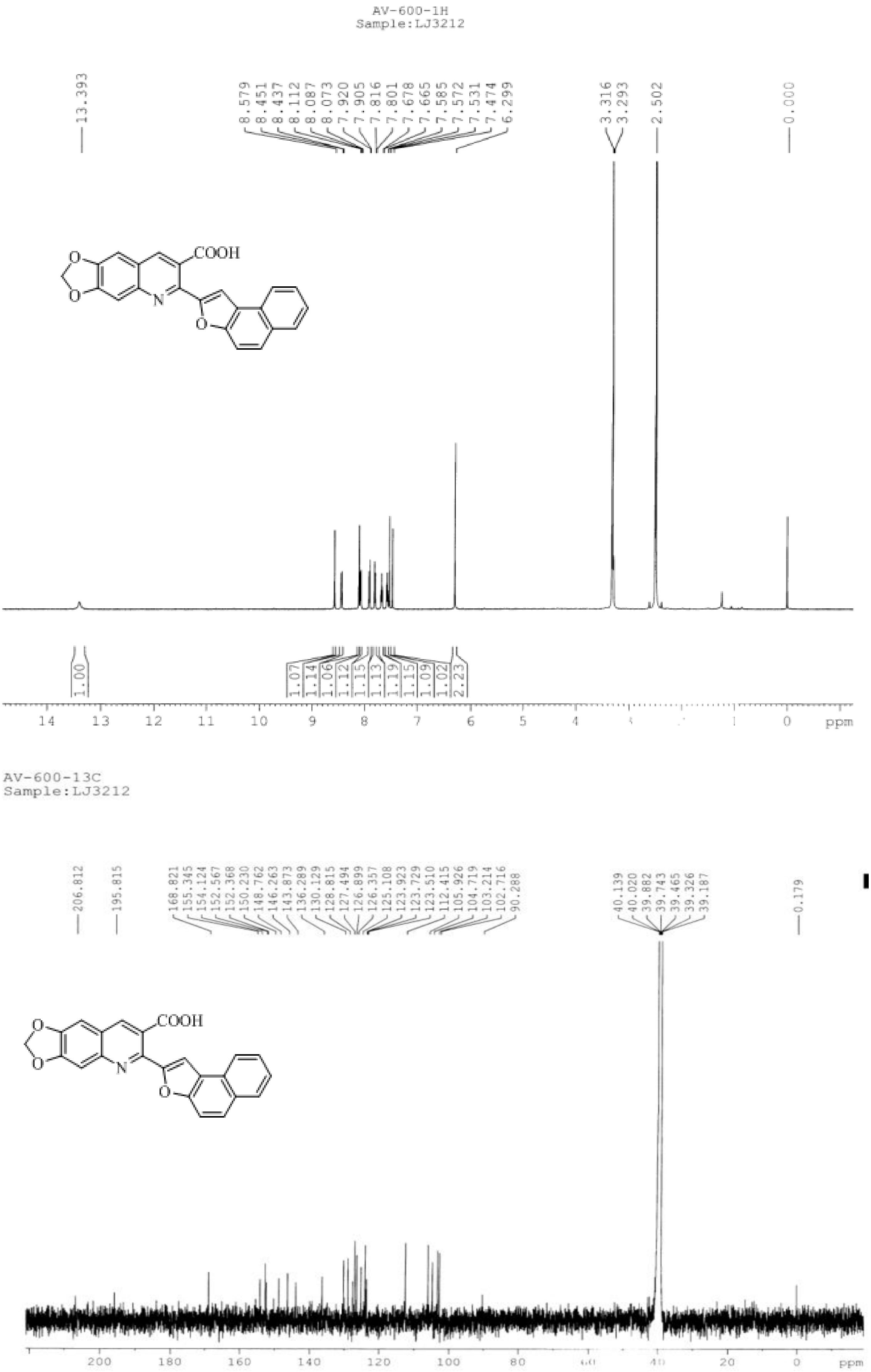

Figure S9: ^{1}H NMR and ^{13}C NMR spectra of **3h**.
